# Blood eosinophils and serum eosinophil cationic protein in patients with acute and chronic urticaria

**DOI:** 10.1155/S0962935196000191

**Published:** 1996-04

**Authors:** G. Di Lorenzo, P. Mansueto, M. Melluso, G. Candore, D. Cigna, M. E. Pellitteri, A. Di Salvo, C. Caruso

**Affiliations:** 1 Cattedra di Medicina Interna II Istituto di Medicina Interna e Geriatria Italy; 2 Servizio di Tipizzazione Tissutale Istituto di Patologia generale Università di Palermo Corso Tukory 211 Palermo 90134 Italy

## Abstract

We have analysed the relationship of blood eosinophil count and serum eosinophil cationic protein (ECP) levels in patients with acute and chronic idiopathic urticaria. The ECP levels and eosinophil counts were measured in the peripheral blood of 15 patients with acute urticaria, 25 with chronic idiopathic urticaria and 10 normal healthy subjects. Blood eosinophil counts and serum ECP levels increased in all patients with acute urticaria. Concerning patients affected by chronic urticaria, taking into account the recrudescence of the disease at the moment of taking the blood sample, only symptomatic patients showed increased eosinophil blood values whereas serum ECP levels were increased both in symptomatic and asymptomatic patients. Furthermore, serum ECP levels in chronic urticaria did not correlate with the peripheral eosinophil counts, as they did in acute urticaria. The results of the present study indicate that eosinophils may play a role in the inflammatory mechanisms in patients with acute and chronic urticaria showing a positive correlation between serum ECP levels and disease activity.


Mediators of Inflammation 5, 113–115 (1996)

### Blood eosinophils and serum eosinophil cationic protein in patients with acute and chronic urticaria

G. Di Lorenzo,[1] P. Mansueto,[1] M. Melluso,[1] G. Candore,[2] D. Cigna,[2] M. E. Pellitteri,[1] A. Di Salvo[1] and C. Caruso[2,CA]

[1]Cattedra di Medicina Interna II, Istituto di Medicina Interna e Geriatria, and [2]Servizio di Tipizzazione Tissutale, Istituto di Patologia generale, Università di Palermo, Corso Tukory 211, 90134 Palermo, Italy. Fax: (+39) 91 6555901

[CA]Corresponding Author

**We have analysed the relationship of blood eosinophil count and serum eosinophil cationic protein (ECP) levels in patients with acute and chronic idiopathic urticaria. The ECP levels and eosinophil counts were measured in the peripheral blood of 15 patients with acute urticaria, 25 with chronic idiopathic urticaria and 10 normal healthy subjects. Blood eosinophil counts and serum ECP levels increased in all patients with acute urticaria. Concerning patients affected by chronic urticaria, taking into account the recrudescence of the disease at the moment of taking the blood sample, only symptomatic patients showed increased eosinophil blood values whereas serum ECP levels were increased both in symptomatic and asymptomatic patients. Furthermore, serum ECP levels in chronic urticaria did not correlate with the peripheral eosinophil counts, as they did in acute urticaria. The results of the present study indicate that eosinophils may play a role in the inflammatory mechanisms in patients with acute and chronic urticaria showing a positive correlation between serum ECP levels and disease activity.**

**Key words**: Acute urticaria, chronic urticaria, ECP, eosinophil

### Introduction

Circumscribed, raised, erythematous, evanescent areas (weals) of oedema that involve the superficial portions of the dermis are known as urticaria. Recurrent episodes of urticaria, characterized by transitory itchy weals, lasting longer than 8 to 12 weeks, are termed chronic.[1] Many cases of acute urticaria are triggered by IgE antibodies to various foods, drugs, inhalants, or other allergens.[2] There is a strong consensus that it is rarely possible to demonstrate IgE antibodies as a cause of chronic urticaria, although in this case autoantibodies against the high-affinity IgE receptor may be involved.[3]

In chronic urticaria, previous studies have demonstrated that an increased number of mast cells, monocytes, T-lymphocytes and eosinophils are present in biopsy specimens.[4–7] Yet the role played by eosinophils in the pathogenesis of skin lesions is not completely understood. The activated eosinophil itself elaborates a variety of inflammatory mediators, including, leukotrienes, the major basic protein, eosinophil cationic protein (ECP), the eosinophil protein X/eosinophil-derived neurotoxin and eosinophil peroxidase.[8] The elaboration of these different mediators is consistent with the hypothesis that the eosinophil can be involved in both allergic and nonallergic mechanisms. Particularly, ECP may affect neurogenic responses and might therefore play a role in itching conditions. Previous studies have provided evidence that ECP may mediate cutaneous tissue damage and, in particular, be involved in chronic idiopathic urticaria.[9–13] Samples from local venous blood in cold urticaria have shown raised levels of histamine and eosinophil chemotactic factor.[14] To gain insight into the role of eosinophils in chronic urticaria, we have analysed the relationship of eosinophils to serum ECP levels in patients with acute and chronic idiopathic urticaria.

### Patients and Methods

*Patients:* Fifteen patients with acute urticaria and 25 patients with chronic idiopathic urticaria took part in the study. Acute and chronic urticaria were respectively defined as weals lasting less than 24 h and as recurrent weals lasting at least 6 weeks or more than 2 months (median 100 weeks). Patients affected by chronic urticaria were split in two groups, taking into account the recrudescence of the disease, at the moment of taking the blood sample. Thirteen patients were symptomatic with daily eruptions, whereas 12 patients were asymptomatic for at least 3 weeks. Patients with urticaria, resulting predominantly

© 1996 Rapid Science Publishers

**Table 1.** Eosinophil blood counts (mean ± S.D.) in healthy subjects and in patients with acute or chronic urticaria

| | *n* | Eosinophils × $10^3/\mu l$ |
|---|---|---|
| Healthy subjects | 10 | 0.190 ± 0.045[a] |
| Acute urticaria | 15 | 0.513 ± 0.211[b] |
| Chronic urticaria | 25 | 0.230 ± 0.090[c] |
| Chronic urticaria (symptomatic) | 13 | 0.270 ± 0.090[d] |
| Chronic urticaria (asymptomatic) | 12 | 0.186 ± 0.069[e] |

a *vs.* b $p = 0.001$

a *vs.* c $p$ = N.S.

a *vs.* d $p = 0.01$

a *vs.* e $p$ = N.S.

b *vs.* c $p = 0.0001$

b *vs.* d $p = 0.001$

b *vs.* e $p = 0.002$

d *vs.* e $p = 0.01$

**Table 2.** Serum ECP values (mean ± S.D.) in healthy subjects and in patients with acute or chronic urticaria

| | *n* | ECP (μg/l) |
|---|---|---|
| Healthy subjects | 10 | 4.04 ± 0.84[a] |
| Acute urticaria | 15 | 35.84 ± 17.25[b] |
| Chronic urticaria | 25 | 25.28 ± 16.08[c] |
| Chronic urticaria (symptomatic) | 13 | 37.77 ± 12.60[d] |
| Chronic urticaria (asymptomatic) | 12 | 11.75 ± 2.68[e] |

a *vs.* b $p = 0.0001$

a *vs.* c $p = 0.0001$

a *vs.* d $p = 0.0001$

a *vs.* e $p = 0.001$

b *vs.* c $p$ = N.S.

b *vs.* d $p$ = N.S.

b *vs.* e $p = 0.0001$

d *vs.* e $p = 0.0001$

from physical causes were excluded. Antihistamine treatment was stopped at least 48 h before the serum samples were collected. None of the patients was taking steroids or immunosuppressive drugs at the time of the study. The control group consisted of ten healthy members of the laboratory staff without urticaria or a history of atopy. All the subjects examined were women. Informed consent was obtained from all participants.

*Eosinophil blood count:* A venous blood sample was collected, and the eosinophils were counted in a Fuchs Rosenthal chamber after staining with eosinophil staining solution.[15]

*Quantification of ECP:* The blood samples were stored for 1 h at room temperature before centrifugation at 1 600 × ***g*** at 4°C for 10 min. Serum was collected and recentrifuged at 1 600 × ***g*** at 4°C for 10 min. The collected samples were stored at –70°C until assay. A double antibody radioimmunoassay test has been used to quantify serum ECP. The assay was performed with commercially available kits purchased from Pharmacia Diagnostics (Uppsala, Sweden), according to manufacturers' instructions. The intra-assay coefficient of variation was less than 70%, and the detection limit was less than 2 μg/l.

*Statistical analysis:* All data are expressed as means ± S.D. Correlations were calculated by linear regression. The values for the different group were compared with Student's *t*-test.

### Results

Table 1 shows mean values of the eosinophil blood counts from the ten normal healthy subjects, the 15 patients with acute urticaria and the 25 patients with chronic urticaria. The mean eosinophil counts were significantly higher both in patients with acute urticaria and in patients with chronic symptomatic urticaria compared to that of the healthy subjects. Patients affected by acute urticaria showed eosinophil count values significantly higher than patients affected by chronic urticaria. No significant difference was found between the healthy subjects and the patients with asymptomatic chronic urticaria.

Diversely, the mean ECP serum concentrations were significantly higher both in patients with acute urticaria and in patients with chronic urticaria compared to that of the healthy subjects; but patients affected by acute urticaria did not show ECP serum concentrations higher than patients affected by chronic urticaria. Taking into account the recrudescence of the chronic urticaria, significant differences were found between the healthy subjects and both symptomatic and asymptomatic patients. Finally, in the asymptomatic chronic patients mean ECP serum concentrations were significantly lower than both in patients affected by acute urticaria and in patients affected by chronic symptomatic urticaria (Table 2).

Eosinophil blood counts and ECP serum concentrations demonstrated a significant positive correlation in patients with acute urticaria (Fig. 1A), whereas eosinophil blood counts and ECP serum concentrations were not related in patients with chronic urticaria analysed either on the whole (Fig. 1B) or on the basis of the recrudescence or not of the disease (data not shown).

### Discussion

The aim of the present study was to investigate the relationships between the blood eosinophil counts and serum ECP levels in patients with acute and chronic urticaria. Blood eosinophil counts and serum ECP levels increased in all patients with acute urticaria. Concerning patients affected by chronic urticaria, taking into account the recrudescence of the disease at the moment of taking the blood sample, only symptomatic patients showed increased eosinophil blood values whereas serum ECP levels were increased both in symptomatic and asymptomatic patients. Furthermore serum ECP levels in chronic urti-

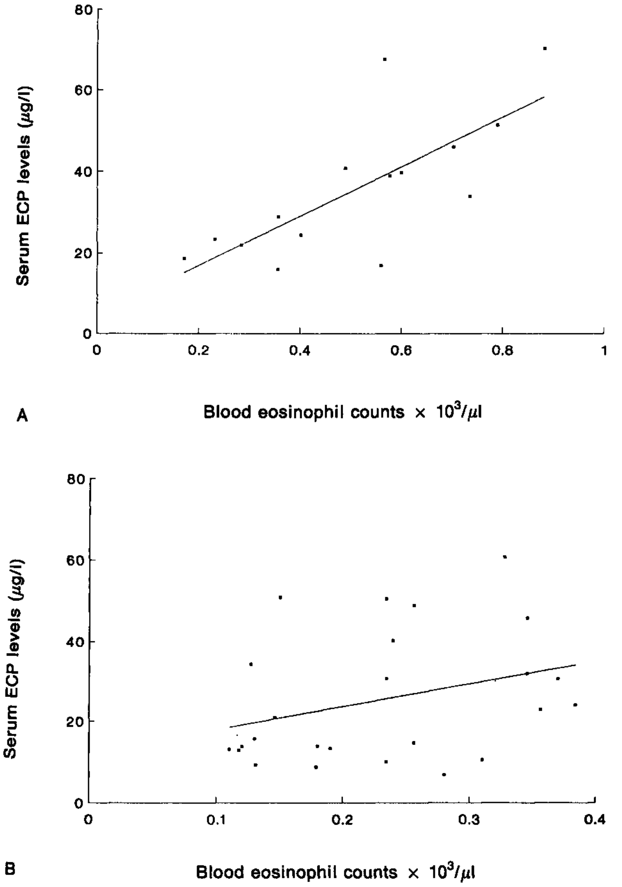

FIG. 1. (A) Correlation between serum ECP values (μg/l) and eosinophil blood counts ($\times$ $10^3$/μl) in 15 patients with acute urticaria ($r = 0.73$; $p = 0.0016$). (B) Correlation between serum ECP values (μg/l) and eosinophil blood counts ($\times$ $10^3$/μl) in 25 patients with chronic urticaria ($r = 0.32$; $p$ = N.S.).

caria did not correlate with the peripheral eosinophil counts, as they did in acute urticaria. However these results indicate that eosinophils may play a pathogenetic role in the inflammatory mechanisms underlying both acute and chronic urticaria.

What are the reasons for the elevated ECP levels in these patients? In order to discuss this question we have to understand the mechanism behind the presence of ECP in serum. Serum levels of ECP are dependent on three factors. One is the number of eosinophils in the blood. The second is the content of ECP of the eosinophil population and the third is the propensity of the eosinophils to secrete their ECP.[16] In acute urticaria the ECP levels are completely paralleled by blood eosinophil counts. This could be due to a parallel change in the production of eosinophils in the bone marrow and in the activation of the eosinophil population. In chronic urticaria discrepancies between blood eosinophil counts and serum ECP levels are found. In this case the induction of eosinophil production and activation are dissociated, as we have observed in asthma patients treated with a long-acting $\beta$2-agonist.[17] In other words, some of the factors involved in the induction and enhancement of eosinophilopoiesis process are not involved in the activation process, and *vice versa*.[16] Thus, depending on the factors produced in the urticaria process, the response of the eosinophil may vary. These variations in the response of the eosinophils probably give rise to the different patterns of blood eosinophil counts and serum ECP levels. Our data show a positive correlation between serum ECP levels and disease activity and support the concept that acute urticaria and chronic urticaria may represent a spectrum of disease activity rather than separate entities.

### References

1. Soter NA. Urticaria: current therapy. *J Allergy Clin Immunol* 1990; **86**: 1009–1014.
2. Mathews KP. Management of urticaria and angioedema. *J Allergy Clin Immunol* 1980; **66**: 347–357.
3. Hide M, Francis DM, Grattan EH, Hakimi J, Kochan JP, Greaves MW. Autoantibodies against the high-affinity IgE receptor as a cause of histamine release in chronic urticaria. *N Engl J Med* 1993; **328**: 1599–1604.
4. Natbony SF, Philips ME, Elias JM, Godfrey HP, Kaplan AP. Histologic studies of chronic idiopathic urticaria. *J Allergy Clin Immunol* 1983; **71**: 177–183.
5. Peters MS, Schroeter AL, Kephart GM, Gelich GJ. Localization of eosinophil granule major basic protein in chronic urticaria. *J Invest Dermatol* 1983; **81**: 39–43.
6. Elias J, Boss E, Kaplan AP. Studies of the cellular infiltrate of chronic idiopathic urticaria: prominent of T-lymphocytes, monocytes, and mast cells. *J Allergy Clin Immunol* 1986; **78**: 914–918.
7. Peters MS, Winkelmann RK, Greaves MW, Kephart GM, Gelich GJ. Extracellular deposition of eosinophil granule major basic protein in pressure urticaria. *J Am Acad Dermatol* 1987; **16**: 513–517.
8. Spry CIF. *Eosinophils: a comprehensive review and guide to the scientific and medical literature.* Oxford: Oxford University Press, 1988.
9. Russel Jones R, Bhogal B, Dash A, Schifferli J. Urticaria and vasculitis: a continuum of histological and immunopathological changes. *Br J Dermatol* 1983; **108**: 695–703.
10. Tai PC, Spry JF, Peterson C, Venge P, Olsson I. Monoclonal antibodies distinguish between storage and secreted forms of eosinophilic cationic protein. *Nature* 1984; **309**: 182–184.
11. Leiferman KM. A current perspective on the role of eosinophils in dermatologogic disease. *J Am Acad Dermatol* 1991; **24**: 1101–1112.
12. Leiferman KM, Norris PG, Murphy GM, Hawk JLM, Winkelmann RK. Evidence for eosinophil degranulation with deposition of granule major basic protein in solar urticaria. *J Am Acad Dermatol* 1989; **21**: 75–80.
13. Juhlin L, Venge P. Eosinophilic cationic protein (ECP) in skin disorders. *Acta Derm Venereal* 1991; **71**: 495–501.
14. Soter NA, Wasserman SI, Austen KF. Cold urticaria release into circulation of histamine and eosinophils chemiotactic factor of anaphylaxis during cold challenge. *New Engl J Med* 1976; **294**: 676–690.
15. Dacie JV, Lewis SM. Eosinophil counts by counting chamber method. In: *Practical Haematology.* Edinburgh: Churchill Livingstone, 1984; 42–43.
16. Venge P. Morning of asthma inflammation by serum measurements of eosinophil cationic protein (ECP). A new clinical approach to asthma management. *Respir Med* 1995; **89**: 1–2.
17. Di Lorenzo G, Morici G, Norrito F, Mansueto P, Melluso M, Purello D'Ambrosio F, Barbagallo Sangiorgi G. Comparison of effects of salmeterol and salbutamol on clinical activity and eosinophil cationic protein serum levels during the pollen season in atopic asthmatics. *Clin Exp Allergy* 1995; **25**: 951–956.

ACKNOWLEDGEMENTS. This work was supported by a grant of the Ministero dell'Università e della Ricerca Scientifica e Tecnologica (60%) to Dr Gabriele Di Lorenzo. The authors would like to thank Professor Joseph Bellanti for his criticism and suggestions.

**Received 6 February 1996; accepted 14 February 1996**
